# Low vision: the patient's perspective

**Published:** 2012

**Authors:** Karin van Dijk

For this issue on low vision, the *Community Eye Health Journal* contacted low vision practitioners in India, Tanzania, Nepal, and Peru to help gather the views of eighteen people attending their low vision clinics. The people varied in age from 14 to 81, and suffered from a range of vision problems including nystagmus, retinitis pigmentosa, diabetic retinopathy, and bilateral aphakia.

The interviewees (or their parents) described how their low vision had affected them before treatment, how their life changed after they received low vision care, and what they felt they still needed.

We hope that these experiences of people with low vision will highlight what is important in a low vision service.

## Before

Before they received low vision care, the adults said they had been unable to do their desired activities, such as driving or reading. They were worried about their vision and had negative feelings, including stress, depression, anger, and frustration. They had also felt dependent on their family, and that they were a burden to the family. The adults had also struggled to accept their condition as being irreversible.

School-age children and young adults said that they had been unable to attend school, had to drop out, or had faced great difficulties in their schooling, such as being unable to take examinations. Some of them had been treated as blind and taught to use Braille.

These young people had also felt very dependent on their families and had to stay home much of the time.

One of the biggest problems they had faced was the way society viewed them. They were victims of bullying, name-calling, and had been accused of pretending to have a problem.

## Care provided

The care provided to both children and adults consisted of training in better use of vision, provision of optical devices, and suggestion of environmental modifications. Specific interventions included:

- Changes such as sitting near the window or using a lamp, sitting near the blackboard, using a stand for better reading/writing position and more comfort, increasing contrast through better light, using a reading slit, and using a cap to reduce glare out of doors
- Giving advice about improving the environment through painting lines or applying tape to improve contrast
- Someone taking the time to clearly explain the person's eye problem and prognosis to him or her
- Counselling, particularly for adults who were able to see before and have lost a lot of their vision. This involved listening, discussing the implications of the vision loss and the effect on their life and emotions, and giving advice if needed.

**Figure:**
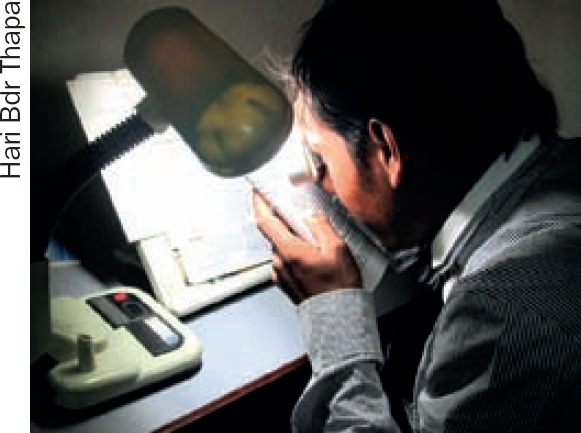
**Damodar BC Nepal (22)** “Society used to view me in a negative way … I used to wonder how I would carry on my life. [But now] there is a positive change in which society views me. Most of the time, I get to hear people say, ‘People with low vision can also do good deeds and can work like normal people.’”

**Figure:**
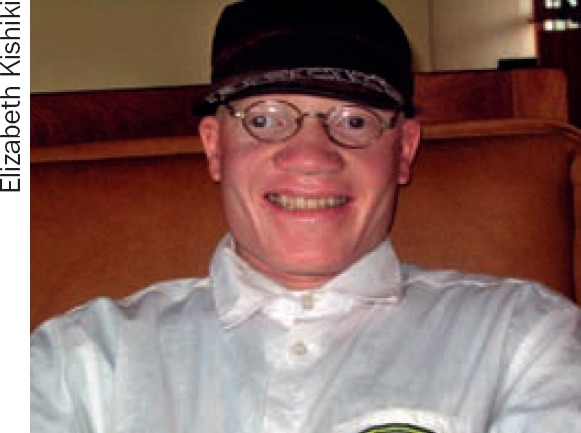
**Mbaraka Omary Tanzania (18)** “I have really started living. With my glasses, I can recognise the faces of my friends and teachers. More importantly, I can watch football and see faces of my favourite stars. With my magnifier, I can read even the smallest letters. I have become a different person now.”

**Figure:**
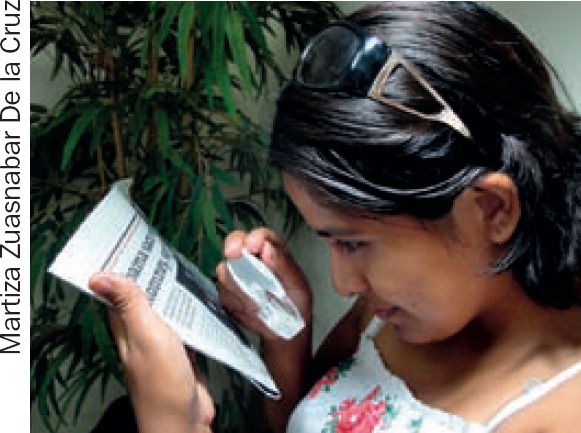
**Maribel Tomateo Falcon Peru (27)** Low vision services helped her to set realistic goals. “The visual rehabilitation helped me a lot, mostly to be aware of my limitations, to accept them and to know up to where I can develop and set my goals.”

**Figure:**
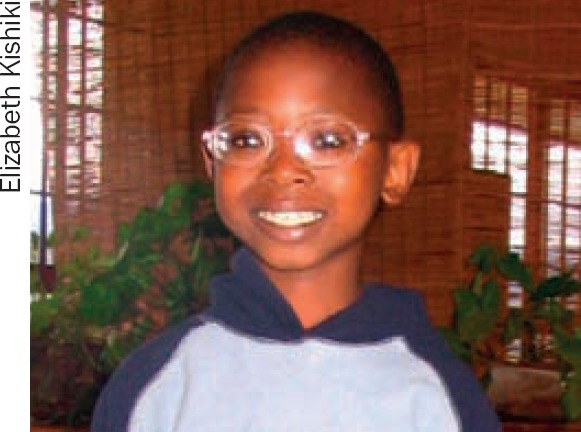
**Abdi Kajembe Tanzania (9)** Thanks to low vision services, school is now a lot easier for him. “With my spectacles, I can sit at my front desk and read well on the blackboard and in books, and I can see people well.”

## Impact

Adults described how low vision services had resulted in the following:

- Greater independence, confidence, courage, hope, and dignity
- A better understanding of the reality of the visual loss.

Children talked about how the low vision service had helped them with the following:

- Starting school
- Doing desired activities, such as reading print, even small print
- Increased independence, for example being able to read the blackboard and learning to write
- Improving the attitudes of peers and teachers “… who now see I can do many things.”
- Better social interaction, for example “…recognising the faces of my friends.”

## What more is needed?

- Some people still lacked the confidence to use their optical devices in public
- Most people also wanted to be informed if there were new technological developments, and hoped for lower prices for software and electronic low vision devices
- Some children did not know enough about their condition and wanted someone to explain it to them in terms they could understand.

In our experience, it is helpful to keep in touch with people who have been helped by low vision services. They can be excellent advocates for the development of better services and may help to convince others with low vision to seek help.

Children who successfully use a low vision device can also inspire other children who are still struggling.

*The interviews were arranged, transcribed, and translated by*:

- Rosario Espinoza, Peru
- Hari Thapa, Nepal
- Elizabeth Kishiki, Tanzania
- *Joseph Eye Hospital LV team, India*.

